# The relationship between chronic lung disease diagnosis and the susceptibility to e-cigarette use in adults: The mediation effects of psychosocial, cognitive influences, and the moderation effect of physiological factors

**DOI:** 10.18332/tid/169741

**Published:** 2023-09-22

**Authors:** Mohammed M. Alqahtani, Abdullah M. M. Alanazi, Hassan Aljohani, Taha T. Ismaeil, Saleh S. Algarni, Tareq F. Alotaibi, Mansour M. Alotaibi, Mohammad Ebrahimi Kalan, Donald H. Lein, Mobarak K. Alqahtani, Khalid S. Alwadeai, Anwar B. Almutairi, Peter S. Hendricks

**Affiliations:** 1 Department of Respiratory Therapy, College of Applied Medical Sciences, King Saud bin Abdulaziz University for Health Sciences, Riyadh, Saudi Arabia; 2 King Abdullah International Medical Research Center, Riyadh, Saudi Arabia; 3 Department of Respiratory Care, King Abdulaziz Medical City, Riyadh, Saudi Arabia; 4 Department of Physical Therapy, College of Applied Medical Sciences, Northern Border University, Arar, Saudi Arabia; 5 School of Health Professions, Eastern Virginia Medical School, Norfolk, United States; 6 Department of Physical Therapy, School of Health Professions, University of Alabama at Birmingham, Birmingham, United States; 7 Department of Rehabilitation Science, College of Applied Medical Sciences, King Saud University, Riyadh, Saudi Arabia; 8 Physical Therapy Department, School of Allied Health, Kuwait University, Kuwait City, Kuwait; 9 Department of Psychiatry, School of Medicine, University of Alabama at Birmingham, Birmingham, United States

**Keywords:** chronic lung disease, susceptibility to using e-cigarettes, cognitive factors, psychosocial factors

## Abstract

**INTRODUCTION:**

There is a paucity of studies on e-cigarette use among adults with chronic lung disease. In the present study, we aimed to assess whether psychosocial or cognitive factors elucidate the relationship between chronic lung disease (CLD) and susceptibility to e-cigarette use and whether the relationship between CLD and e-cigarette use is conditional on the presence of respiratory symptoms.

**METHODS:**

We recruited adults aged ≥18 years in Alabama with CLD from university medical clinics (n=140) and individuals without CLD (n=123 as a reference group). Information on sociodemographics, susceptibility to e-cigarette use, psychosocial factors, and cognitive factors were collected. Mediation analysis was used to assess whether the psychosocial factors or cognitive factors explained the association between CLD and susceptibility to using e-cigarettes, and moderation analysis was conducted to determine if respiratory factors would change the association between CLD and susceptibility to e-cigarette use.

**RESULTS:**

Psychosocial factors (stress, depression, anxiety) and e-cigarette positive expectancy were notably high among individuals with CLD. Having CLD was associated with a lower likelihood of susceptibility to e-cigarette use. Higher levels of stress, being a smoker, boredom, taste/sensorimotor manipulation, and social facilitation were associated with higher odds of susceptibility to using e-cigarettes among individuals with CLD. Mediation analysis indicated a statistically significant indirect effect of CLD on the susceptibility to using e-cigarettes through stress and boredom reduction. We did not find a statistically significant interaction between CLD and respiratory symptoms affecting susceptibility to using e-cigarettes.

**CONCLUSIONS:**

Individuals with CLD often exhibit stress, depression, and a positive view of e-cigarettes but are generally less inclined to use them. Stress, smoking habits, boredom, taste, and social influence can increase their susceptibility to e-cigarette use. Our findings call for further exploration to evaluate the temporal relationship between CLD status, psychosocial factors, cognitive factors, and susceptibility to using e-cigarettes.

**TRIAL REGISTRATION:**

The study was registered on ClinicalTrials.gov, on 5 November 2019. Identifier: NCT04151784

## INTRODUCTION

Electronic cigarettes (also known as e-cigarettes or vapes) are battery-powered devices that vaporize liquid solutions containing nicotine, flavoring, and other substances such as vegetable glycerin and propylene glycol^1-4^. Although e-cigarettes are not risk-free, they have been touted as a safer alternative to traditional cigarettes^5,6^.

An e-cigarette is not solely water vapor, and its use may harm human health. For instance, the aerosol inhalation that e-cigarettes produce may still pose a pulmonary risk by increasing reactivity, obstruction, and inflammation^7^. Researchers also report that e-cigarette use is associated with increased respiratory symptoms, poorer lung function, negative pulmonary effects, and other adverse outcomes^8,9^. Further, it is documented that e-cigarette exposure may increase the risk of cardiovascular disease^7,10^.

Despite the evidence concerning health risks, the prevalence of e-cigarette use is high. For example, in the United States, in 2020, it was estimated that more than 5.66 million adults reported currently vaping (2.3%; 95% CI: 2.2–2.4)^11^. When examined among different age groups, young adults (18–34 years) demonstrated the greatest e-cigarette use at 8.3%, and use generally decreased with age: 35–44 years (4.3%), 45–54 years (5.2%), 55–64 years (2.6%), and ≥65 years (0.8%)^12^.

Furthermore, some studies have demonstrated a notable percentage of e-cigarette use among adults within certain vulnerable populations, such as those with chronic lung disease (CLD)^13-16^. Therefore, elucidating the factors that lead to e-cigarette use, especially among those with CLD, is crucial for informing the design of prevention and treatment strategies. These should include a harm reduction approach or an approach that discourages use patterns that maintain an addiction to tobacco cigarettes among individuals with chronic lung disease.

Modifiable behaviors that may influence susceptibility to using e-cigarettes, specifically among those with CLD, have been infrequently studied. It is known that people with CLD are more likely to suffer from mental health conditions such as depression, stress, and anxiety^17-19^. In fact, several studies have found support for the negative reinforcement model of drug addiction (sometimes referred to as the ‘self-medication’ model), in which individuals with mental health issues use substances such as tobacco to regulate some of the associated psychological symptoms^20,21^. Evidence also indicates an association between mental health conditions and susceptibility to using e-cigarettes^22^. However, as noted above, little is known about the relationship between mental health conditions and susceptibility to e-cigarette use in individuals with CLD.

Another potential reason for e-cigarette use may be perceived positive outcome expectations for e-cigarette use. Individuals may expect e-cigarette use will produce positive effects, including reducing negative moods, providing sensory satisfaction, and enhancing positive moods and social interaction^23,24^. Positive outcome expectations are associated with an increased likelihood of e-cigarette use in the past month and more willingness to use an e-cigarette in the future^25^. Moreover, recent work suggests that positive e-cigarette outcome expectations are related to greater perceived benefits from e-cigarette use^24^. Positive e-cigarette outcome expectations have also been associated with lower intentions to quit e-cigarettes^23,26^. Other studies suggest that the increase in e-cigarette use has been driven, in part, by beliefs that e-cigarettes are less harmful than combustible cigarettes^27,28^. Indeed, the expected positive consequences of substance use are well-supported mechanisms explaining smoking motivation and behavior and may also explain e-cigarette use^29^. In fact, contemporary theory posits that expectations serve as a final common pathway involved in the substance use decision-making process^30^. Regarding susceptibility to using e-cigarettes, however, research on expectation and beliefs toward e-cigarette use among vulnerable populations is sparse. Research on perceived beliefs toward e-cigarette use among those with CLD is even more so.

The goal of the present study is to determine whether the association between CLD status and susceptibility to using e-cigarettes is mediated by one’s mental health condition or by cognitive factors. We hypothesized that: 1) CLD would be associated with poorer mental health, which would, in turn, be associated with a greater susceptibility to e-cigarette use; 2) CLD would be associated with cognitive factors (positive outcome expectation), which would in turn be associated with a greater susceptibility to e-cigarette use; 3) the association between CLD status and susceptibility to using e-cigarettes would be moderated by the presence or absence of respiratory symptoms.

## METHODS

Data were collected from January 2020 to March 2021. Two groups aged ≥18 years were recruited. The first group (n=141) were individuals with a diagnosis of CLD [obstructive lung diseases based on the International Classification of Diseases, Tenth Revision (ICD-10)] who sought CLD-related medical management at an outpatient healthcare clinic. These individuals were recruited in person through a questionnaire packet given to anyone who expressed interest in the study, and treated at the University of Alabama at Birmingham (UAB) outpatient pulmonary clinic (the Kirklin Clinic of UAB Hospital) or the UAB Lung Health Center, who expressed interest in participating in the study. Additional individuals with a diagnosis of CLD in the UAB clinical databases were recruited by email or mail. These individuals received a recruitment flyer that included information about the study and a personalized link to a web-based survey option.

The second group (control group) was composed of individuals without CLD (n=123), and they served as the control/reference group. The individuals in this group were enrolled via purposive convenience sampling using posted recruitment flyers on the UAB campus. Individuals who did not have CLD and expressed interest in participating were emailed a personalized link to the web-based survey option.

Individuals who were currently using e-cigarettes in both groups were not allowed to participate in the study because our variable of interest was susceptibility to using e-cigarettes. Those who took part in the survey received a $15 incentive to compensate them for their participation. Ethical approval was given by the University of Alabama at Birmingham’s institutional review board. Finally, this study’s protocol was registered with ClinicalTrials.gov before data analysis was performed (NCT04151784).

### Dependent variable

*Susceptibility to e-cigarette use*

The questions used to assess susceptibility to e-cigarette use were from previous studies^31-33^, which adopted validated measures of susceptibility to smoking combustible cigarettes. Each participant’s susceptibility to using e-cigarettes was ascertained based on their answers to the following four questions: ‘Do you think that you will use an e-cigarette soon?’, ‘Do you think that, in the future, you might experiment with e-cigarettes?’, ‘Do you think you will use an e-cigarette in the next year?’ and ‘If one of your best friends were to offer you an e-cigarette, would you smoke it?’. Possible responses were: ‘definitely not’, ‘probably not’, ‘probably yes’, and ‘definitely yes’. A summary measure of susceptibility to e-cigarette use was created based on responses to these four questions, such that a participant was classified as not susceptible to e-cigarette use if they responded ‘definitely not’ to all four items or susceptible to e-cigarette use if they responded: ‘probably not’, ‘probably yes’, or ‘definitely yes’, to any of the four items.

### Independent variable

*CLD status*

Participants in the reference group (no CLD) were coded as ‘0’; participants with any type of CLD were coded as ‘1’^33^.

### Mediating variables

*Cognitive factors*

A second version of the Brief Smoking Consequences Questionnaire-Adult (BSCQ-A) was used in this study. This was made up of items specific to e-cigarettes. The BSCQ-A measures expectations on the same ten scales: negative affect reduction, stimulation/state enhancement, health risks, taste/sensorimotor manipulation, social facilitation, weight control, craving/addiction, negative physical feelings, boredom reduction, and negative social impression^29^. Cronbach’s alpha reliabilities of the e-cigarette-specific BSCQ-A also were comparable to the tobacco-specific BSCQ-A and ranged from 0.67 to 0.88.

*Depression, stress, and anxiety*

Questions from the Depression, Anxiety, and Stress Scale (DASS-21) were used to assess depression, anxiety, and stress. The DASS-21 is a clinical assessment that measures the three related states of depression, anxiety, and stress. It has 21 questions. The internal consistency of the DASS-21 scales was acceptable (depression: α=0.829; anxiety: α=0.778; stress: α=0.871)^34^.

### Covariates

Seven self-reported covariates were collected and incorporated in the analyses: age (18–81 years); gender (male or female); race (African American/Black, Asian/Filipino, Pacific Islander/Native Hawaiian, Caucasian/White, American Indian/Alaskan Native, more than one ethnic group, not known, and other); ethnicity (Hispanic, and non-Hispanic); education level (less than 12 years of education, high school, Associate in Arts (AA) or in Science (AS) degree, other vocational program, BA or BSc, MA or MSc, PhD, MD, or JD); and tobacco use (never, former, and current users)^33^.

### Statistical analysis

Data analysis was done using SPSS version 28. Independent t-tests were used to compare continuous variables, and chi-squared tests were used to compare nominal variables between groups. Mediation pathway analysis was performed using PROCESS Macro Indirect effects, and standard errors were computed using 10000 bootstrapped samples. A p<0.05 was considered statistically significant for all analyses.

## RESULTS

Our study demonstrated that individuals with CLD were more likely to be female, non-Hispanic, Caucasian/White, married, though not living with a spouse/partner, older, employed, high school graduates, have an annual income <$10000, heterosexual, and cigarette smokers (Table 1).

**Table 1:** Sociodemographic characteristic of study participants

*Characteristics*	*Without chronic lung disease* *(N=123)* *n (%)*	*With chronic lung disease* *(N=140)* *n (%)*	*χ* ^2^ *p*
**Gender**			2.640 0.267
Female	56 (45.5)	72 (51.4)	
Male	54 (43.9)	50 (35.7)	
Other	1 (0.8)	0 (0)	
Missing	12 (9.8)	18 (12.9)	
**Hispanic/Latino descent**			5.911 0.05*
Yes	10 (8.1)	3 (2.1)	
No	100 (81.3)	119 (85.0)	
Not Sure	1 (0.8)	0 (0)	
Missing	12 (9.8)	18 (12.9)	
**Race**			13.034 0.034*
African American/Black	19 (15.4)	29 (20.7)	
Asian/Filipino	7 (5.7)	2 (1.4)	
Pacific Islander/Native Hawaiian	0 (0)	0 (0)	
Caucasian/White	73 (59.3)	86 (61.1)	
American Indian/Alaskan Native	0	2 (1.4)	
More than one ethnic group	3 (2.4)	1 (0.7)	
Not known	1 (0.8)	0 (0)	
Other	8 (6.5)	2 (1.4)	
Missing	12 (9.8)	18 (12.9)	
**Marital status**			15.389 0.002*
Married	46 (37.4)	43 (30.7)	
Widowed	2 (1.6)	6 (4.3)	
Divorced/separated	10 (8.1)	33 (23.6)	
Single/never married	52 (42.3)	40 (28.6)	
Missing	12 (10.6)	18 (12.9)	
**Live with a spouse or partner**			0.130 0.718
Yes	54 (43.9)	57 (40.7)	
No	56 (45.5)	65 (46.4)	
Missing	13 (10.6)	18 (12.9)	
**Age** (years), mean ± SD	35.85 ± 13.48	48.01 ± 16.69	t= -6.114 <0.001*
**Employment status**			39.906 <0.001*
Employed	48 (39.0)	44 (31.4)	
Unemployed	18 (14.6)	30 (21.4)	
Retired	6 (4.9)	33 (23.6)	
Full-time homemaker	3 (2.4)	7 (5.0)	
Student (not employed)	35 (28.5)	8 (5.7)	
Missing	13 (10.6)	18 (12.9)	
**Education level**			9.764 0.082
Less than 12 years	9 (7.3)	14 (10.0)	
High school, GED	25 (20.3)	33 (23.6)	
AA, AS, other vocational program	11 (8.9)	24 (17.1)	
BA, BSc etc.	30 (24.4)	28 (20.0)	
MA, MSc etc.	26 (21.1)	15 (10.7)	
PhD, MD, JD etc.	10 (8.1)	8 (5.7)	
Missing	12 (9.8)	18 (12.9)	
**Income** ($)			9.504 0.147
<10000	34 (27.6)	33 (23.6)	
11000–20000	22 (17.9)	29 (20.7)	
21000–30000	17 (13.8)	8 (5.7)	
31000–40000	29 (23.6)	30 (21.4)	
41000–50000	3 (2.4)	6 (4.3)	
51000–60000	0	0	
61000–70000	3 (2.4)	6 (4.3)	
71000–80000	3 (2.4)	10 (7.1)	
81000–90000	0 (0)	0 (0)	
91000–100000	0 (0)	0 (0)	
>100000	0 (0)	0 (0)	
Missing	12 (9.8)	18 (12.9)	
**Sexual orientation**			0.769 0.681
Homosexual/gay/lesbian	3 (2.4)	6 (4.3)	
Heterosexual	100 (81.3)	109 (77.9)	
Bisexual	7 (5.7)	7 (5.0)	
Missing	13 (10.6)	18 (12.9)	
**Lifetime smoking status**			10.078 0.002*
No	61 (49.6)	43 (30.7)	
Yes	50 (40.7)	82 (58.6)	
Missing	12 (9.8)	15 (10.7)	
**Current smoking status**			5.283 0.071
Every day	23 (18.7)	30 (21.4)	
Some days	12 (9.8)	11 (7.9)	
Not at all	16 (13.0)	42 (30.0)	
Missing	72 (58.5)	57 (40.7)	

* p<0.05.

As shown in Table 2, multivariable modeling revealed that, compared with not having CLD, having these diseases was associated with a lower likelihood of susceptibility to e-cigarette use. Additionally, higher levels of stress, smoking status, boredom, taste/sensorimotor manipulation, and social facilitation were associated with higher odds of susceptibility to using e-cigarettes.

**Table 2:** Multivariable models of susceptibility to e-cigarette use predicted by cognitive factors and psychosocial factors, and the interaction (moderation) effects with chronic lung disease with respect to susceptibility to using e-cigarette use

*Chronic lung disease*	*B (95% CI)*	*SE*	*t*	*p*
No	1.069 (0.281–1.857)	0.402	2.66	0.008*
Yes	-1.069 (-1.857 – -0.281)	0.402	-2.66	0.008*
Stress	0.234 (0.099–0.368)	0.068	3.40	0.001*
Depression	-0.109 (-0.228–0.009)	0.054	-1.81	0.071
Anxiety	-0.065 (-0.201–0.070)	0.069	-0.94	0.346
Negative affect reduction	-0.038 (-0.128–0.052)	0.046	-0.82	0.410
Stimulation/state enhancement	0.017 (-0.119–0.153)	0.069	0.24	0.807
Health risks	0.0132 (-0.079–0.106)	0.047	0.28	0.781
Taste/sensorimotor manipulation	0.144 (0.059–0.229)	0.043	3.32	0.001*
Social facilitation	0.097 (0.016–0.178)	0.041	2.35	0.019*
Appetite/weight control	-0.039 (-0.109–0.029)	0.035	-1.12	0.263
Craving/addiction	0.084 (-0.033–0.201)	0.059	1.41	0.157
Negative physical feelings	-0.093 (-0.192–0.006)	0.051	-1.84	0.066
Boredom reduction	-0.135 (-0.265 – -0.004)	0.067	-2.02	0.043
Social impression	-0.022 (-0.085–0.041)	0.032	-0.69	0.49
**Constant**	4.105 (1.616–6.594)	1.269	3.23	0.001

Models were adjusted for smoking behavior, age, gender, education level, ethnicity, and race.

* p<0.05.

### Mediation analysis

*Mental health conditions*

We first introduced all mental health conditions (stress, depression, and anxiety) as parallel mediators of the relationships between having CLD and susceptibility to e-cigarette use based on 10000 bootstrapped samples. We then found that the direct effect of CLD status on odds of susceptibility to using e-cigarette use was statistically significant (β= -1.053; 95% CI: -1.781 – -0.326).

CLD status was associated with a higher level of stress (β=1.886; 95% CI: 0.843– 3.288), which in turn was a significant predictor of susceptibility to using e-cigarettes (β=0.163; 95% CI: 0.044–0.283). Consistent with mediation, the specific indirect effect of CLD status on the susceptibility to using e-cigarettes through stress was statistically significant (β=0.308; 95% CI: 0.040–0.809).

In addition, CLD status was associated with a higher level of depression (β=1.424; 95% CI: 0.007–2.842), which was not a significant predictor of susceptibility to using e-cigarettes (β= -0.099; 95% CI: -0.209– 0.011). Consistent with mediation, the indirect effect of CLD status on susceptibility to using e-cigarettes through depression was statistically insignificant (β= -0.141; 95% CI: -0.490–0.037).

**Figure 1:**
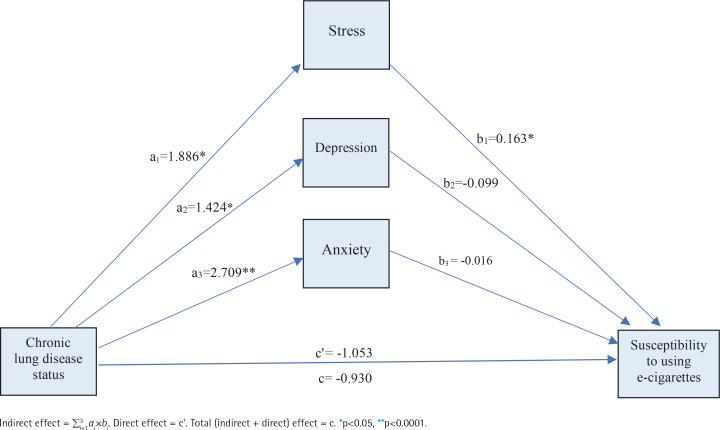
Mediated relationship between chronic lung disease status and susceptibility to using e-cigarette with mental health conditions (stress, depression, anxiety) as the mediator

Finally, CLD status was associated with a greater level of anxiety (β=2.709; 95% CI: 1.407–4.010), which in turn was not a significant predictor of susceptibility to using e-cigarettes (β= -0.016; 95% CI: -0.143–0.111). Consistent with mediation, the indirect effect of CLD status on susceptibility to using e-cigarette use through anxiety was statistically insignificant (β= -0.044; 95% CI: -0.488–0.346) ( Figure 1).

*Cognitive factors*

We first introduced all cognitive factors (negative affect reduction, stimulation/state enhancement, health risks, taste/sensorimotor manipulation, social facilitation, appetite/weight control, craving/addiction, negative physical feelings, boredom reduction, and social impression) as parallel mediators of the relationships between the presence of CLD and susceptibility to e-cigarette use based on 10000 bootstrapped samples, and the direct effect of CLD status on odds of susceptibility to e-cigarette use was statistically significant (β= -1.108; 95% CI: -1.939 – -0.277).

CLD status was associated with a lower negative affect reduction (β= -2.167; 95% CI: -4.697–0.362), which in turn was not a significant predictor of susceptibility to using e-cigarettes (β= -0.047; 95% CI: -0.129–0.025). Consistent with mediation, the indirect effect of CLD status on susceptibility to using e-cigarettes through negative affect reduction was statistically insignificant (β=0.101; 95% CI: -0.112–0.560). Moreover, CLD status was negatively associated with stimulation/state enhancement (β= -0.937; 95% CI: -2.431–0.558), which in turn was not a significant predictor of susceptibility to using e-cigarettes (β=0.019; 95% CI: -0.112–0.150). Consistent with mediation, the indirect effect of CLD status on susceptibility to using e-cigarettes through stimulation/state enhancement was statistically insignificant (β= -0.018; 95% CI: -0.291–0.169).

CLD status was associated negatively with health risks (β= -0.651; 95% CI: -2.678–1.276), which in turn was not a significant predictor of susceptibility to using e-cigarettes (β= -0.003; 95% CI: -0.090–0.083). Consistent with mediation, the indirect effect of CLD status on susceptibility to using e-cigarettes through health risks was statistically insignificant (β=0.002; 95% CI: -0.119–0.136).

CLD status was associated negatively with taste/sensorimotor manipulation (β= -1.211; 95% CI: -3.774–1.352), which in turn was not a significant predictor of susceptibility to using e-cigarettes (β=0.136; 95% CI: 0.053–0.218). Consistent with mediation, the indirect effect of CLD status on susceptibility to using e-cigarettes through taste/sensorimotor manipulation was statistically insignificant (β= -0.164; 95% CI: -0.746–0.215). In addition to the above, CLD status was associated negatively with social facilitation (β= -1.854; 95% CI: -3.893–0.184), which in turn was a significant predictor of susceptibility to using e-cigarettes (β=0.096; 95% CI: 0.018–0.175). Consistent with mediation, the indirect effect of CLD status on susceptibility to using e-cigarettes through social facilitation was statistically insignificant (β= -0.179; 95% CI: -0.583–0.029). Moreover, CLD status was associated negatively with appetite/weight control (β= -1.471; 95% CI: -3.558–0.615), which in turn was a significant predictor of susceptibility to using e-cigarettes (β= -0.027; 95% CI: -0.092–0.038). Consistent with mediation, the indirect effect of CLD status on susceptibility to using e-cigarettes through appetite/weight control was statistically insignificant (β=0.040; 95% CI: -0.114–0.256). CLD status was also associated negatively with craving/addiction (β= -1.277; 95% CI: -2.963–0.409), which in turn was a significant predictor of susceptibility to using e-cigarettes (β=0.090; 95% CI: -0.021–0.202). Consistent with mediation, the indirect effect of CLD status on the likelihood of current e-cigarette use through craving/addiction control was statistically insignificant (β= -0.115; 95% CI: -0.455–0.100).

**Figure 2:**
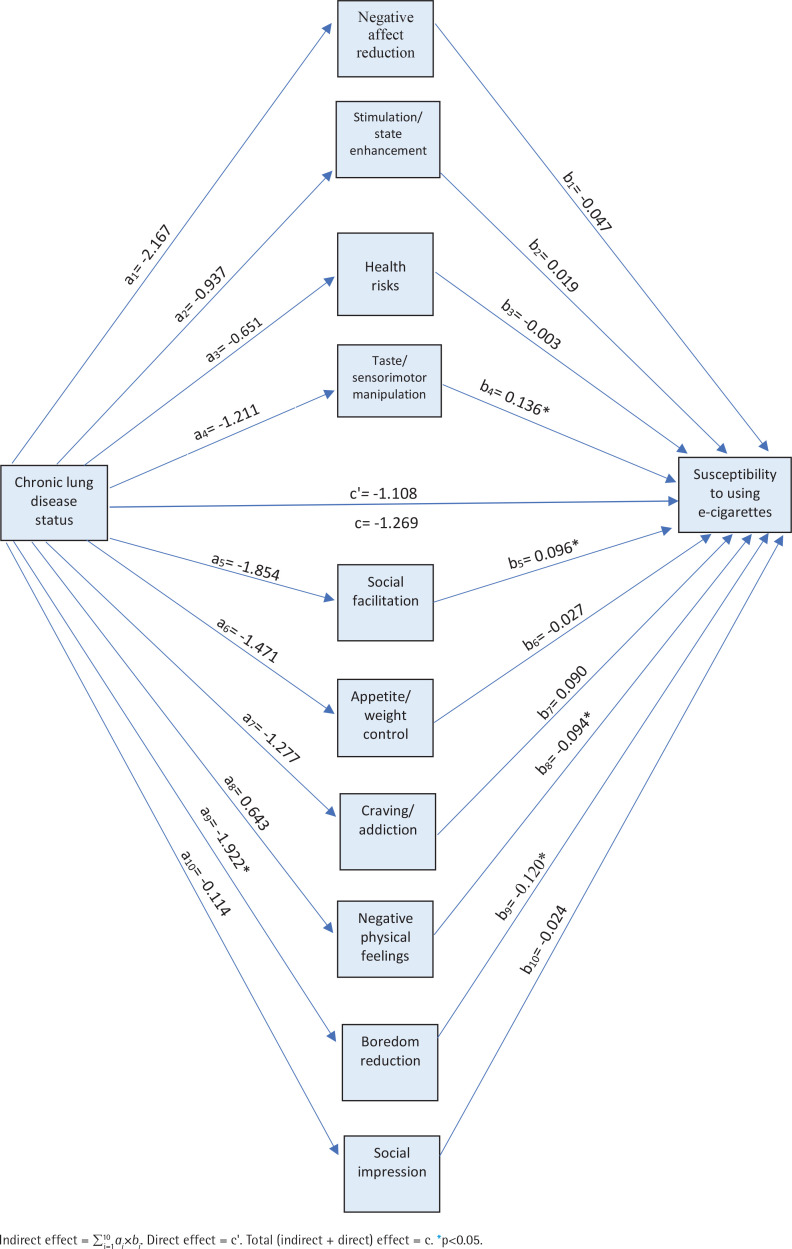
Mediated relationship between chronic lung disease status and susceptibility to using e-cigarette with cognitive factors (stress, depression, anxiety) as the mediator

CLD status was associated negatively with negative physical feelings (β= -0.643; 95% CI: -1.064– 2.350), which in turn was a significant predictor of susceptibility to using e-cigarettes (β= -0.094; 95% CI: -0.186 – -0.001). Consistent with mediation, the indirect effect of CLD status on the likelihood of current e-cigarette use through negative physical feelings was statistically insignificant (β= -0.060; 95% CI: -0.314–0.120). CLD status was associated negatively with boredom reduction (β= -1.922; 95% CI: -3.674 – -0.170), which in turn was a significant predictor of susceptibility to using e-cigarettes (β= -0.120; 95% CI: -0.242–0.003). Consistent with mediation, the indirect effect of CLD status on the likelihood of current e-cigarette use through boredom reduction was statistically significant (β=0.230; 95% CI: -0.025–0.760). Furthermore, CLD status was associated negatively with social impression (β= -0.114; 95% CI: -2.919–2.692), which in turn was an insignificant predictor of susceptibility to using e-cigarettes (β= -0.024; 95% CI: -0.084–0.036). Consistent with mediation, the indirect effect of CLD status on the likelihood of current e-cigarette use through social impression was statistically significant (β=0.003; 95 % CI: -0.141–0.146) (Figure 2).

### Moderation analysis

There was no statistically significant interaction between CLD and respiratory symptoms regarding the odds of susceptibility to using e-cigarettes (β= -0.104; 95 % CI : -0.517–0.30).

## DISCUSSION

The aim of the present study was to assess whether the association between CLD status and susceptibility to e-cigarette use was mediated by mental health conditions/or cognitive factors. Our study showed that mental health conditions and some positive beliefs toward e-cigarettes were higher among individuals with CLD. E-cigarette use has also spurred concerns about possible dire health consequences, particularly for individuals who suffer from lung conditions^35^. The use of e-cigarettes among adults with CLD should be curtailed. This implies that an increased understanding of the mechanisms linking CLD status, cognitive factors, and psychosocial factors to susceptibility to using e-cigarettes is critical for developing preventive and treatment strategies that improve e-cigarette use abstinence rates among individuals with CLD who also suffer from concomitant mental health conditions as well as having misperceptions regarding e-cigarettes.

Psychosocial factors (i.e. stress, depression, and anxiety) were noticeably high among individuals with CLD, as found in earlier studies^17,36,37^. Further, stress contributing to susceptibility to e-cigarette use may be elucidated by the negative reinforcement model of drug addiction. This model suggests that escape or avoidance of negative affect encompasses a motivational foundation for compelling drug use^21^. The possible reason for that is people with CLD may use e-cigarettes to decrease the mental health symptoms that come with their lung health condition^20,21^. Our findings also accord with earlier findings that recognized a link between poor mental health and e-cigarette use^38-40^. Notably, the influence of stress on the relationship between CLD and susceptibility to e-cigarettes accords with our previous work, which demonstrated that more severe mental health conditions account for the increased prevalence of e-cigarette use in young adults with asthma and adults with CLD^14,37^.

The present findings suggest that positive expectations for e-cigarette use may be an influential individual difference variable to consider. Results indicated that beliefs about the positive effects of using e-cigarettes might be more prominent among individuals with CLDs, which in turn, may further validate persistent use and reinforce their maladaptive cognitive processes, such as experiencing greater perceived benefits from e-cigarette use despite the observed adverse effects of use^41^, compared to those without CLD. The present findings suggest a possible need to contextualize positive expectations for e-cigarettes among individuals with CLD.

### Limitations

Our study has some limitations. First, with the cross-sectional study design, we cannot sufficiently elucidate the relationship between CLD status, psychosocial factors, cognitive factors, physiological factors, and susceptibility to using e-cigarettes. Second, response bias and recall bias may hide the correlation between variables despite controlling for the potential confounding variables. In addition, we only evaluated susceptibility to e-cigarette use and did not assess the information on actual e-cigarette use, which may have some positive effects on the lungs of established smokers who completely switch to e-cigarette use. Third, we enrolled individuals with a clinical diagnosis of CLD (obstructive lung disease). We did not include individuals with other CLDs, such as restrictive lung diseases. Fifth, we did not control for the other chronic health conditions that may affect the results of this study, such as diabetes, cancer, and cardiovascular disease. Fourth, a relatively small sample size may limit the generalization of our results. Despite these limitations, results from this study can serve as the basis for additional research to guide regulatory efforts for e-cigarette use among adults with CLD and add to the existing literature on e-cigarettes.

### Implications

Mental health referrals should be taken into consideration when healthcare providers deal with individuals who are seeking tobacco treatment and have CLD. Preventive measures should be implemented in individuals with/without CLD; they should be aware of how mental health conditions may lead to substance use/abuse, such as e-cigarettes, and they should be aware and informed about how e-cigarette misperceptions may lead to e-cigarette use. Additionally, this research may impart an insight into cognitive factors related to e-cigarette use expectations that can be targeted with health communication to prevent/decrease e-cigarette use among individuals with CLD. Future studies are needed to determine changes among susceptible individuals over extended periods and to determine the temporal relationship between CLD status, susceptibility to using e-cigarettes, mental health conditions, and cognitive factors.

## CONCLUSIONS

Mental health conditions and some cognitive factors appear to be predictors of susceptibility to using e-cigarettes. However, stress and boredom reduction seem to mediate the relationship between CLD and the susceptibility to using e-cigarettes. Tailored public health messages are crucial to targeting psychosocial and cognitive factors to prevent and/or decrease e-cigarette use among individuals with CLD^33^.

## Data Availability

The data supporting this research are available from the authors on reasonable request.
